# Supplementary material for: Translational Regulation by hnRNP H/F Is Essential for the Proliferation and Survival of Glioblastoma
Source: Cancers (Basel). 2022 Mar 2;14(5):1283. doi: 10.3390/cancers14051283 (PMC8909726; doi:10.3390/cancers14051283)
Supplement: Supplementary file 1 [file cancers-14-01283-s001.zip › cancers-1607588-supplementary.pdf]

cancers MDPI

Supplementary Material: Translational Regulation by hnRNP H/F Is Essential for the Proliferation and Survival of Glioblastoma

Morgane Le Bras, Noah Gorelick, Sylvain Pautet, Betty Tyler, Stéphane Manenti, Nicolas Skuli, Stefania Millevoi and Anne Cammas

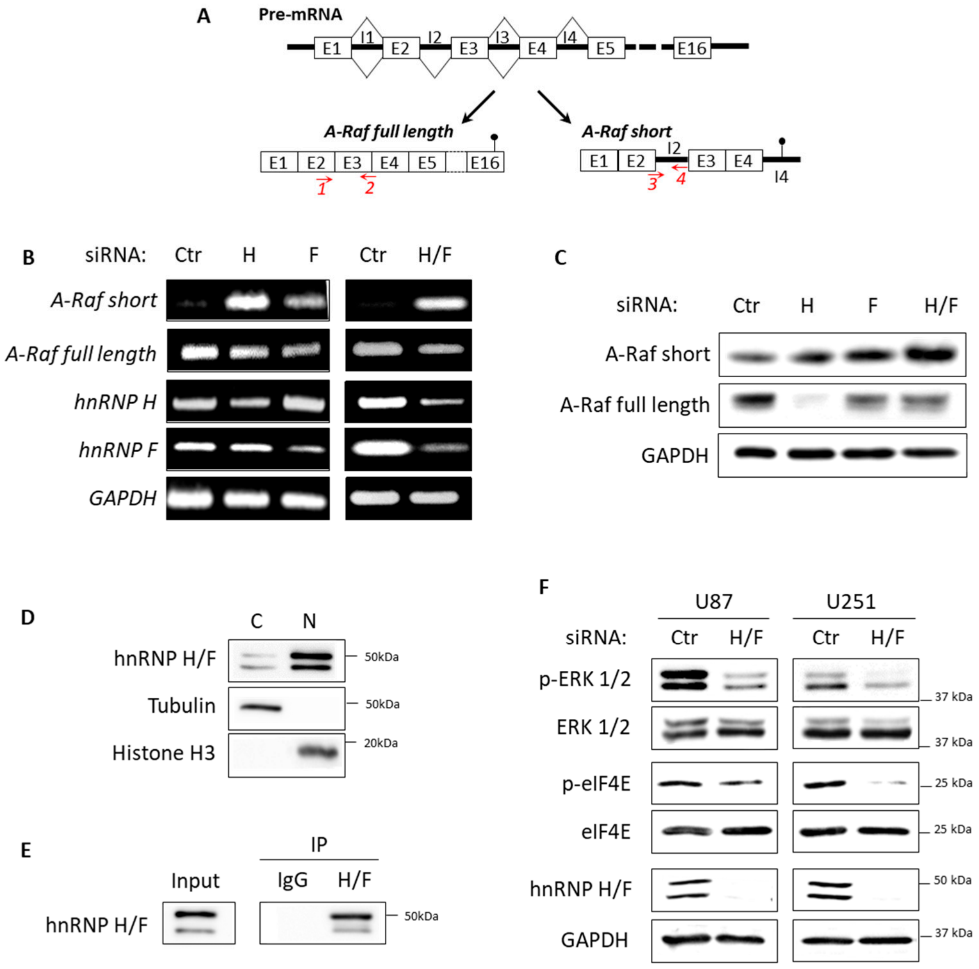

Figure S1. hnRNP H/F impact on eIF4E phosphorylation and A-Raf splicing in different GBM cells. **(A)** Diagram of *A-Raf* pre-mRNA alternative splicing generating the dominant-negative *A-Raf short* isoform (detected with the primer pair 3-4 represented by red arrows) or the *A-Raf full length* (detected with the primer pair 1-2 represented by red arrows). Introns are represented with black lines (I), exons with empty boxes (E) and stop codons with black circles. **(B)** RT-PCR using specific primers for *A-Raf short* isoform on total RNA extracted from LN18 cells treated with 2.5 nM of siRNA control (siCtr), siRNAs against hnRNP H and/or hnRNP F (siH, siF or siH/F) for 72 h. Shown is a representative result from $n = 3$ independent experiments. **(C)** Western blot analysis of A-Raf short and A-Raf full length protein levels in LN18 cells treated with 2.5 nM of siRNA control (siCtr), siRNAs against hnRNP H and hnRNP F (siH/F) for 72 h. **(D)** Subcellular fractionation of U87 cell line, followed by western blot analysis of hnRNP H/F, tubulin (cytosolic marker) and histone H3 (nuclear marker). Nuclear (N) and cytosolic fractions (C). Shown is a representative result from $n = 3$ independent experiments. **(E)** IP of RNA-protein complexes in nuclear extracts from U87 cells with the hnRNP H/F antibody, followed by western blot analysis. Shown is a representative result from n=3 independent experiments. **(F)** Western blot analysis of phospho-ERK 1/2 (p-ERK 1/2), ERK 1/2, phospho-eIF4E (p-eIF4E) and eIF4E expression in U87 and U251 cells treated with 2.5 nM of siRNA control (siCtr), siRNAs against hnRNP H and hnRNP F (siH/F) for 72 h.

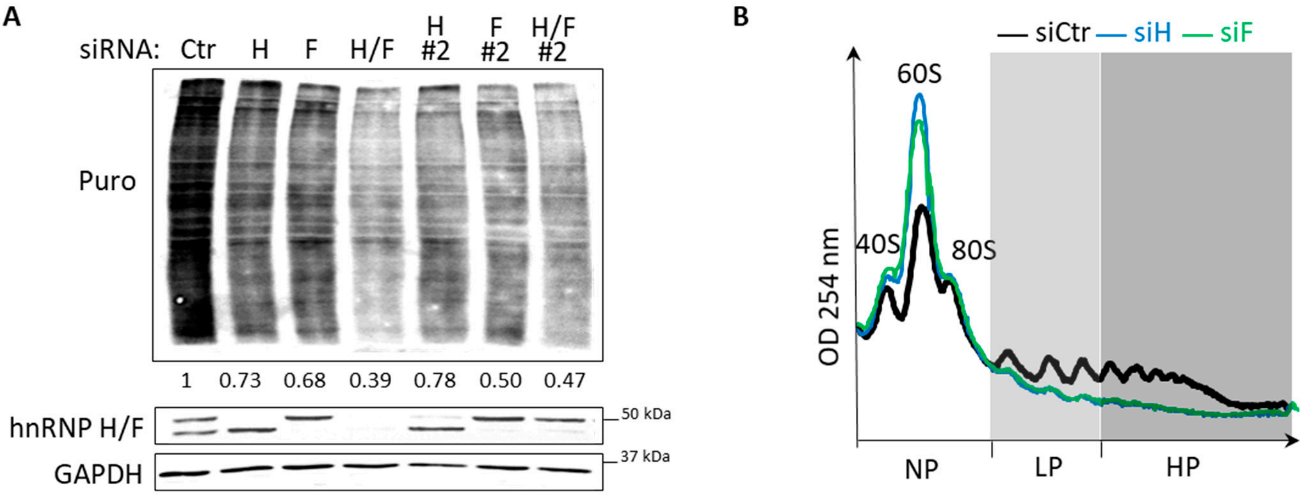

Figure S2. hnRNP H/F effect on translation is confirmed using two gene specific siRNAs on SUnSET assay and polysome profiling experiment. (**A**) *De novo* protein synthesis analysis by SUnSET assay in LN18 cells treated with 2.5 nM siRNA control (siCtr), and 2 different siRNAs against hnRNP H and/or hnRNP F (siH, siF, siH/F, siH#2, siF#2, siH/F#2) for 72 h, followed by western blot analysis of the incorporated puromycin, hnRNP H/F and GAPDH. Puromycin levels were normalized to GAPDH protein level and to the siCtr condition. Quantification of the puromycin signal is indicated at the bottom of the gel. Shown is a representative result from n = 3 independent experiments. (**B**) Polysome profile of LN18 cells treated with 2.5 nM siRNA control (siCtr) and siRNAs against hnRNP H or hnRNP F (siH, siF) for 72 h. The positions of the 40S, 60S and 80S ribosomal subunits and non- (NP), light (LP) and heavy (HP) polysomal fractions are indicated.

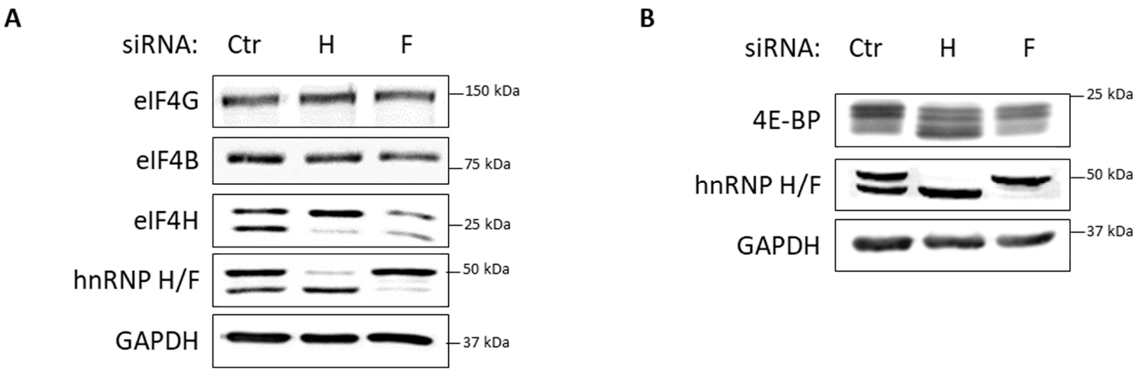

Figure S3. hnRNP H/F knockdown controls eIF4H isoform expression and 4E-BP phosphorylation in GBM cells. **(A, B)** Western Blot analysis of (**A**) eIF4G, eIF4B, eIF4H and (**B**) 4E-BP expression in LN18 cells treated with 2.5 nM of siRNA control (siCtr), siRNAs against hnRNP H and hnRNP F (siH and siF) for 72 h. Shown is a representative result from n = 3 independent experiments.

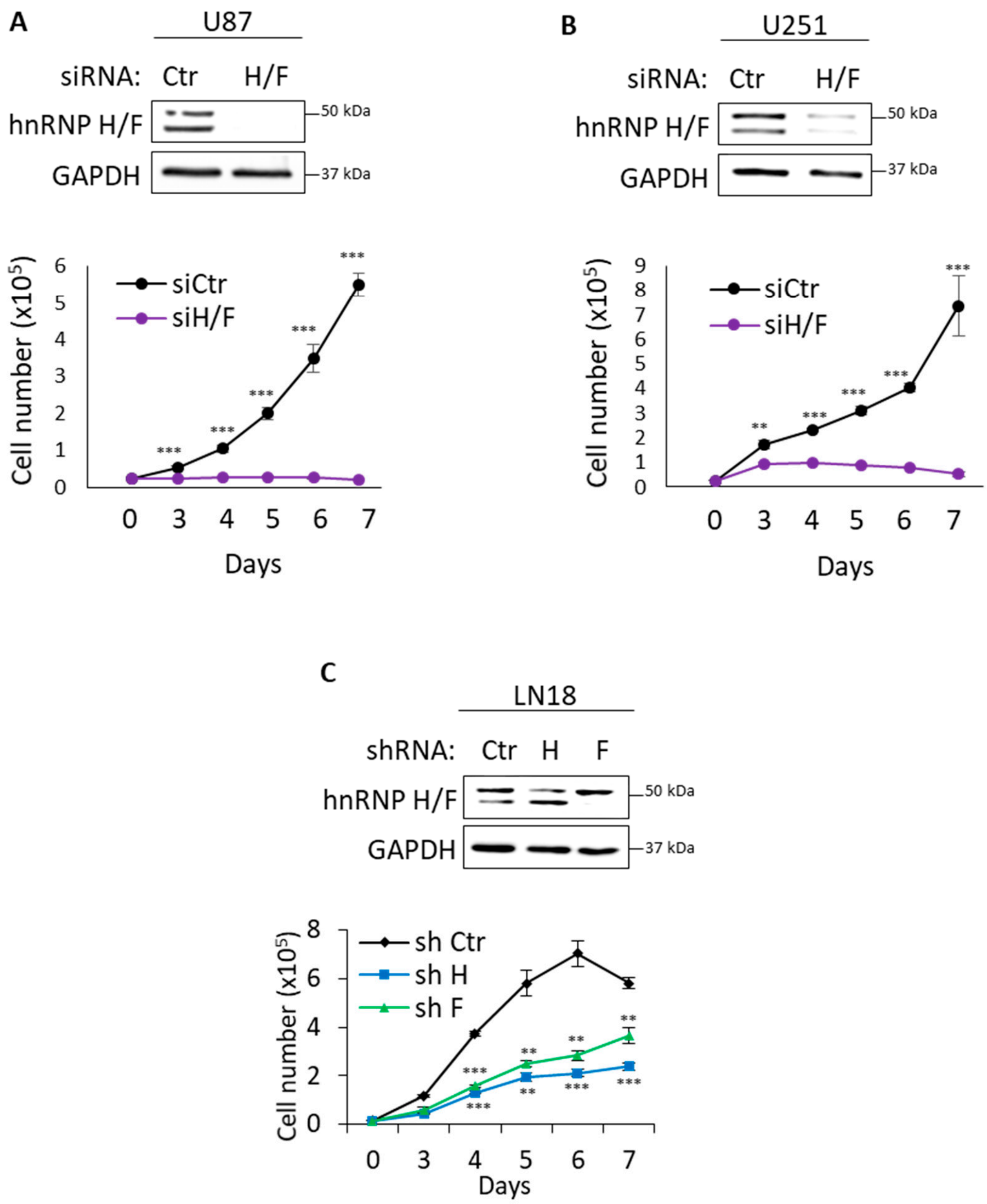

Figure S4. hnRNP H/F knockdown inhibits proliferation in different GBM cells. **(A, B, C)** Proliferation assay and western blot analysis of **(A) U87,** **(B)** U251 or **(C)** LN18 cells treated with 2.5 nM of siRNA control (siCtr), siRNAs against hnRNP H and hnRNP F (siH/F) for 72 h, or shRNAs against hnRNP H or hnRNP F (shH or shF). Data are presented as mean values ± SEM of n=3 independent experiments, **$p < 0.005$ and ***$p < 0.0005$ (two-sided paired t-test).

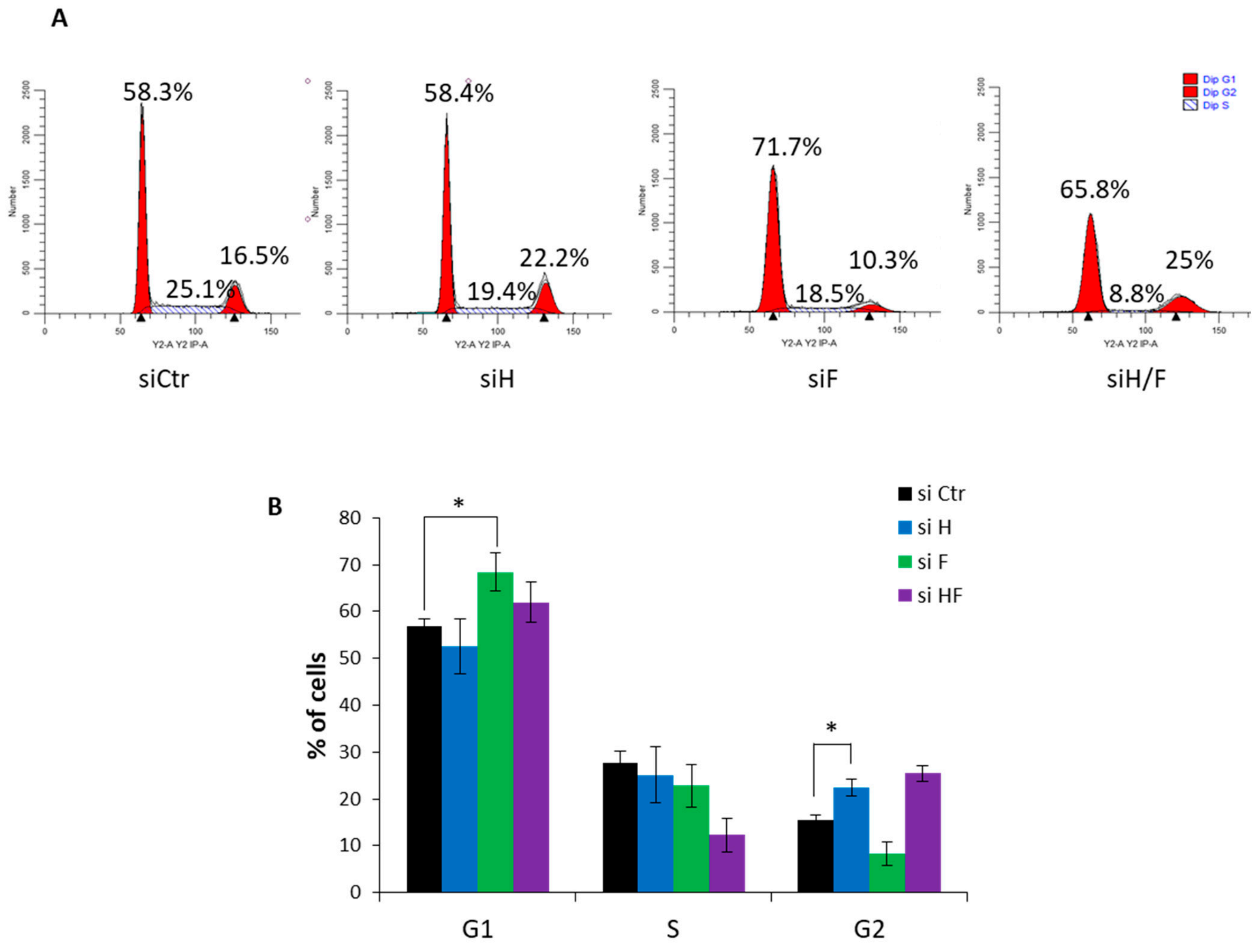

Figure S5. hnRNP H/F depletion induces glioblastoma cell cycle arrest. **(A)** Representative flow cytometry images after propidium iodide (PI) staining of LN18 cells treated with 2.5 nM of siRNA control (siCtr), siRNAs against hnRNP H and/or hnRNP F (siH, siF or siH/F) for 72 h. **(B)** Quantification of LN18 cell distribution in the G1, S or G2 cell cycle phases from (A). Data are presented as mean values ± SEM of n=3 for siH and siF or n = 2 for siH/F independent experiments, *$p < 0.05$ (two-sided paired t-test).

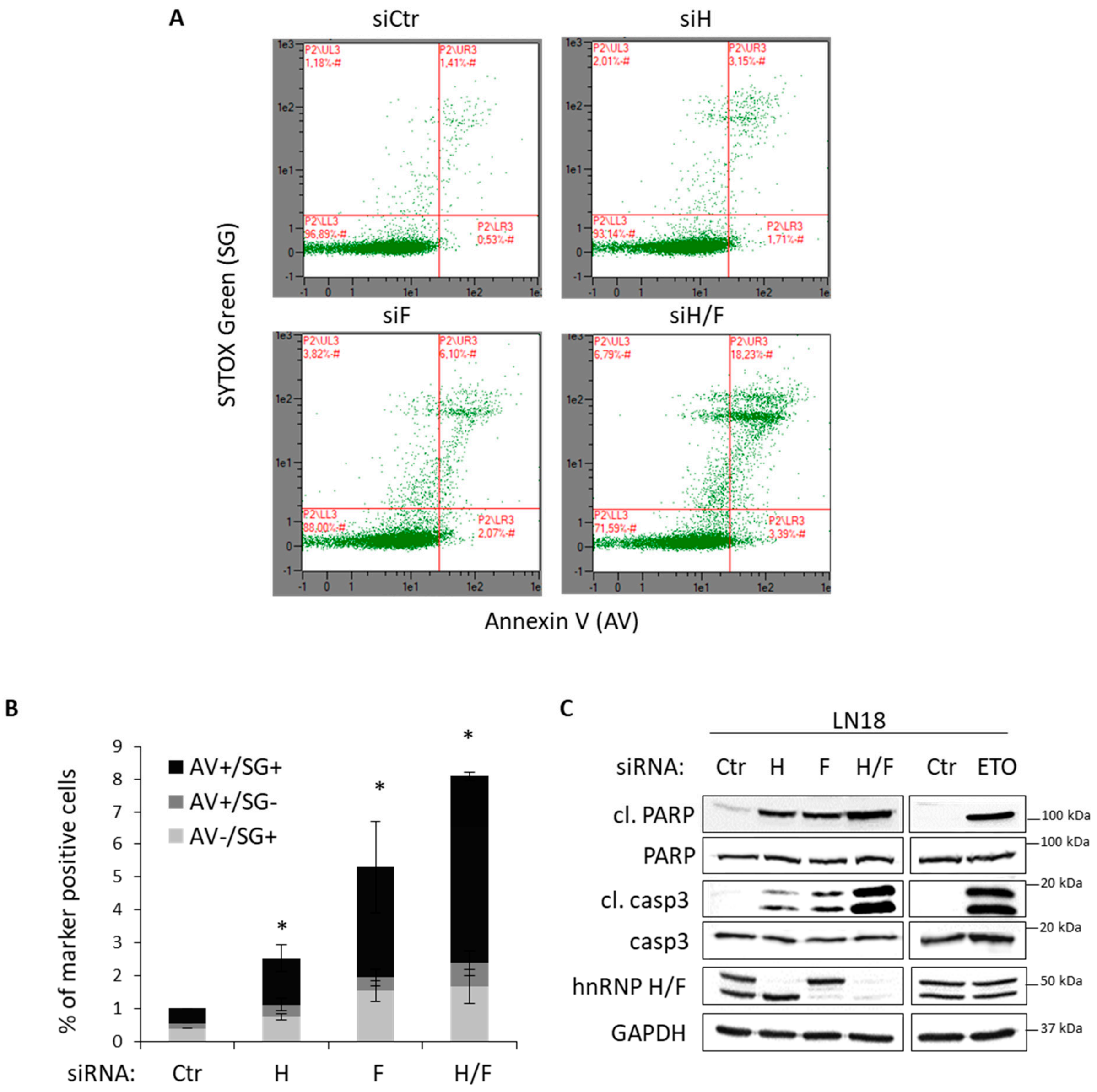

Figure S6. hnRNP H/F depletion increases GBM cell apoptosis**. (A)** Dot plot representing the percentage of both apoptotic and necrotic LN18 cells treated with 2.5 nM of siRNA control (siCtr), siRNAs against hnRNP H and/or hnRNP F (siH, siF and siH/F) for 72 h and analyzed by flow cytometry and Annexin V (AV)/ SYTOX Green (SG) double staining. **(B)** Quantification of Annexin V (AV)/SYTOX Green (SG) combination positive cells from (A). Data are presented as mean values ± SEM of n=3 independent experiments, *p<0.05 (two-sided paired t-test). **(C)** Western Blot analysis of cleaved PARP (cl. PARP), PARP, cleaved caspase 3 (cl. casp3) and caspase 3 (casp3) expression in LN18 cells treated with 2.5 nM of siRNA control (siCtr), siRNAs against hnRNP H and/or hnRNP F (siH, siF and siH/F) for 72 h. Etoposide (ETO) treatment at 100 µg/mL for 72 h constitutes a positive control.

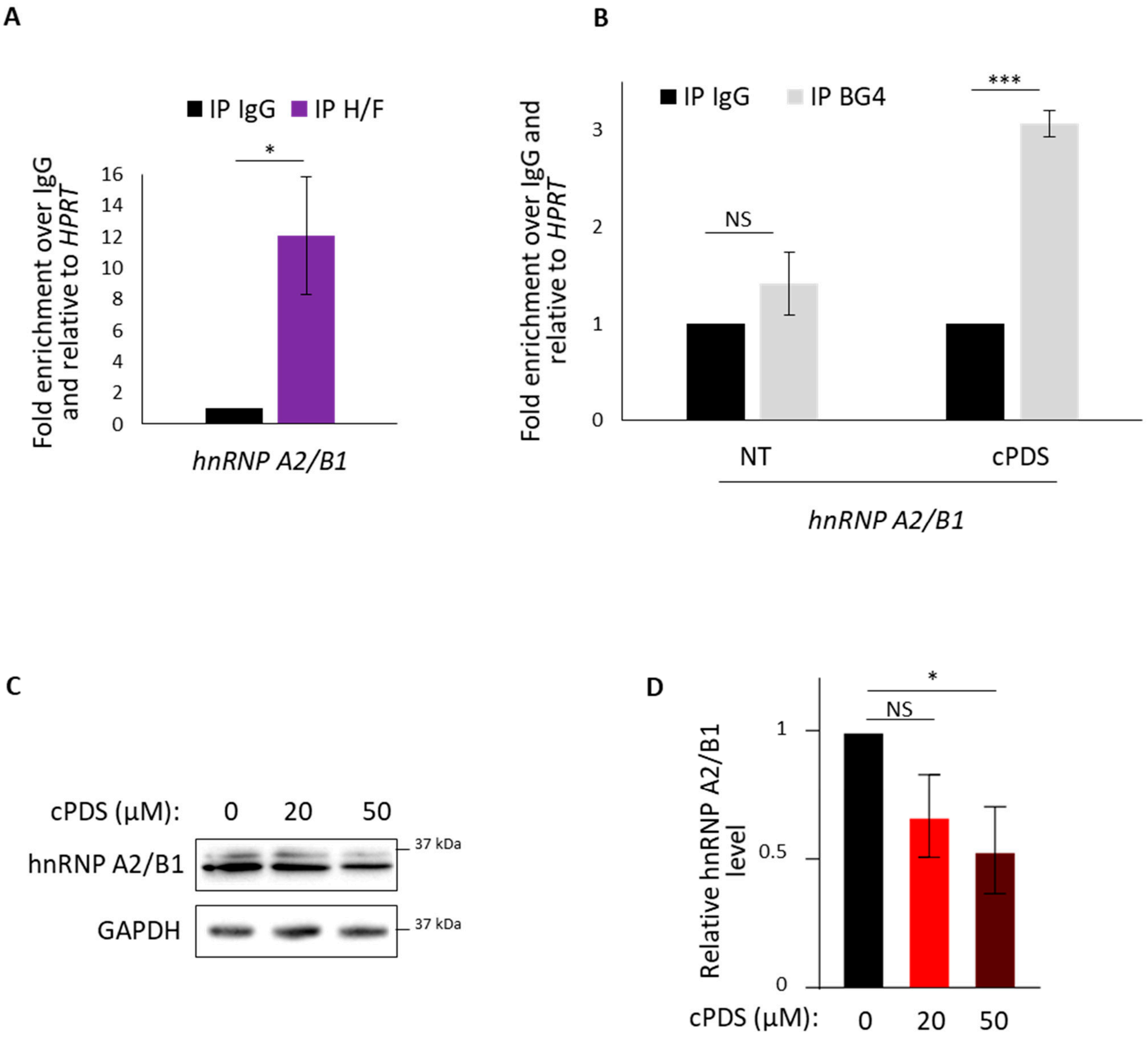

Figure S7. *hnRNP A2/B1* mRNA interact with hnRNP H/F, contain an RG4 and are affected by RG4-stabilization. **(A)** Immunoprecipitation of RNA-protein complexes (RIP) performed on U87 cytoplasmic cell extracts using control IgG (IP IgG) or hnRNP H/F (IP H/F) antibody, followed by RT-qPCR analysis of *hnRNP A2/B1* and *HPRT* mRNAs. Data are plotted as mean values ± SEM of $n = 3$ independent experiments. **(B)** Immunoprecipitation (IP) of RNA-protein complexes (RIP), using control IgG (IP IgG) or BG4 (IP BG4) antibody, performed on cytoplasmic extracts from U87 cells untreated (NT) or treated with 20 µM carboxypyridostatin (cPDS) for 2 h, followed by RT–qPCR analysis of *hnRNP A2/B1* and *HPRT* mRNAs. Data are plotted as mean values ± SEM of $n = 3$ independent experiments. **(C)** Western Blot analysis of hnRNP A2/B1 expression in LN18 cells treated with dose scale of carboxypyridostatin (cPDS) for 48 h. Shown is a representative result from n=3 independent experiments. **(D)** hnRNP A2/B1 protein levels in (C) were normalized to GAPDH protein levels and plotted relatively to the non-treated condition (0 µM cPDS). For panels (A), (B) and (D), data are presented as mean values ± SEM of $n = 3$ independent experiments, *$p < 0.05$, ***$p < 0.0005$ and NS: Non-Significant (two-sided paired t-test).

Figure S8 Original Western Blot

Whole Western Blot for Figure 1C.

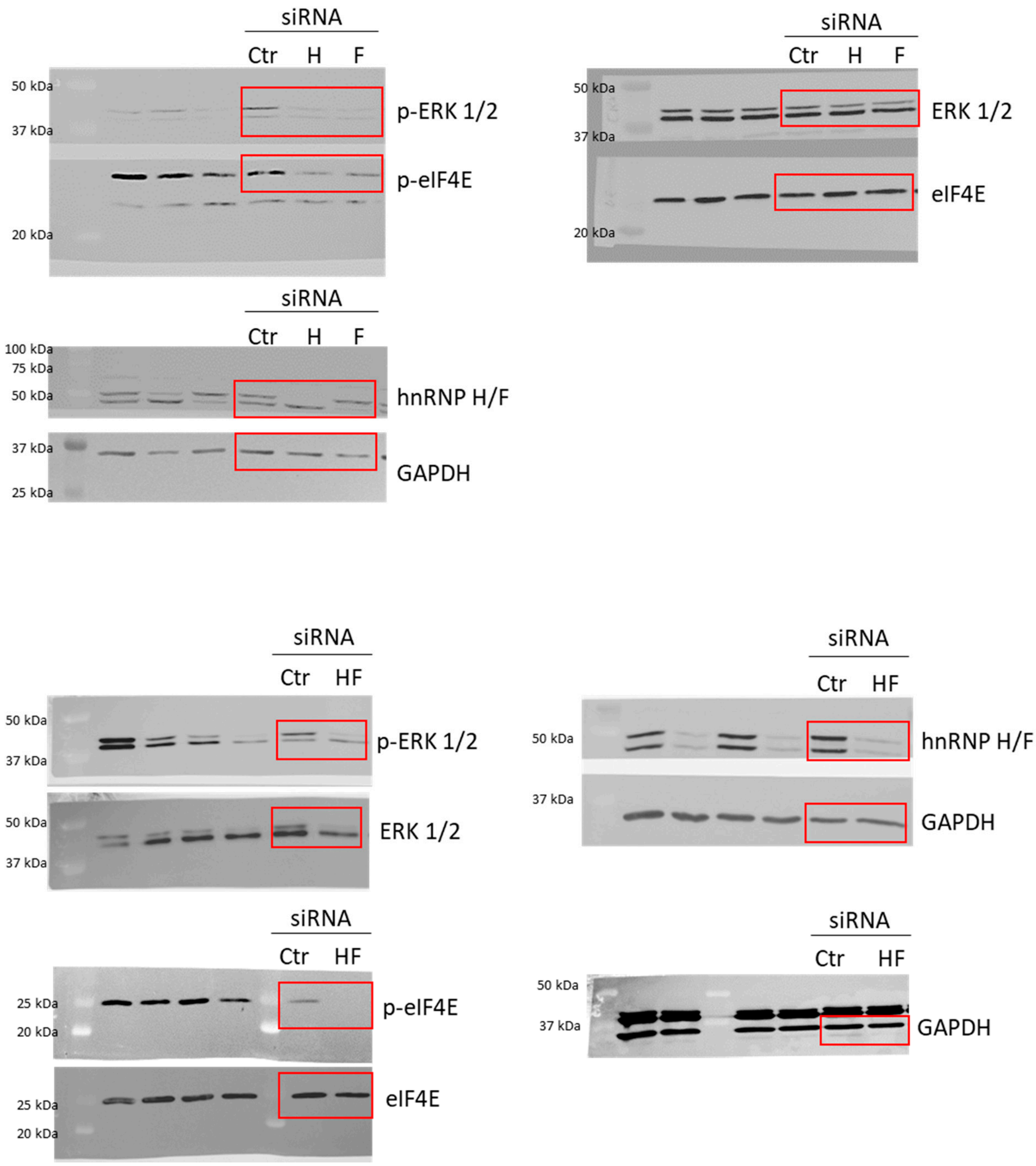

Whole Western Blot for Figure 2C.

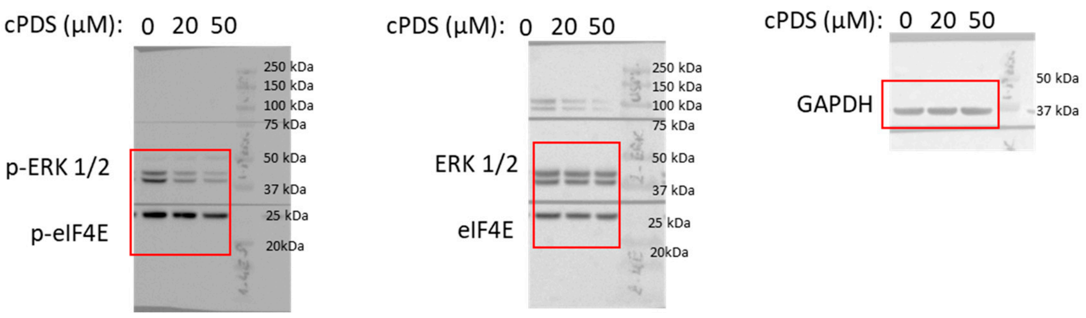

Whole Western Blot for Figure 3A.

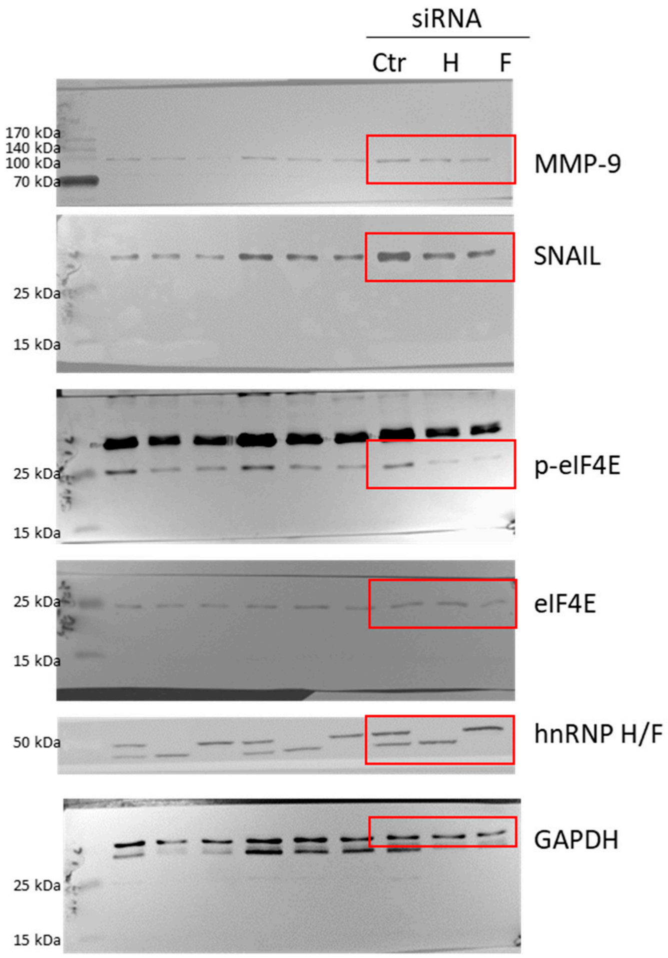

Whole Western Blot for Figure 3C.

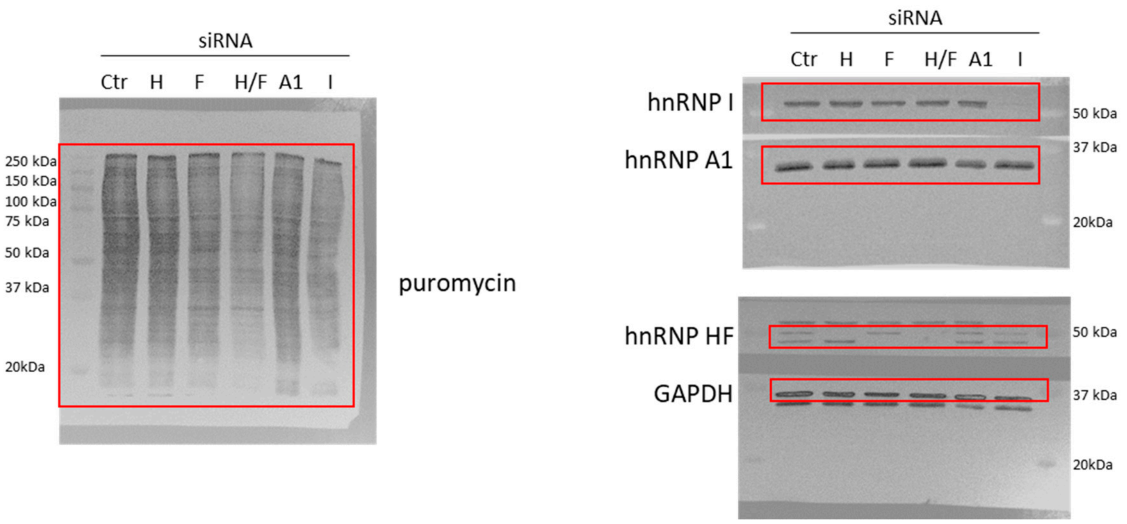

Whole Western Blot for Figure 5.

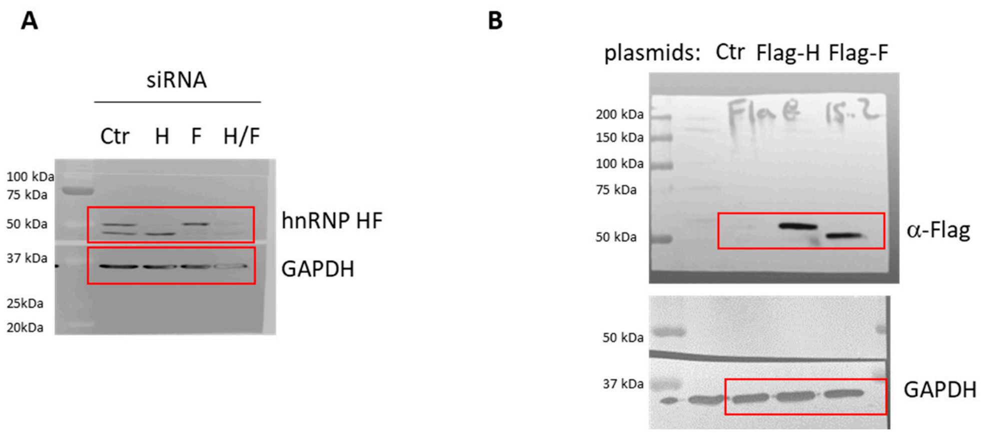

Whole Western Blot for Figure S1F

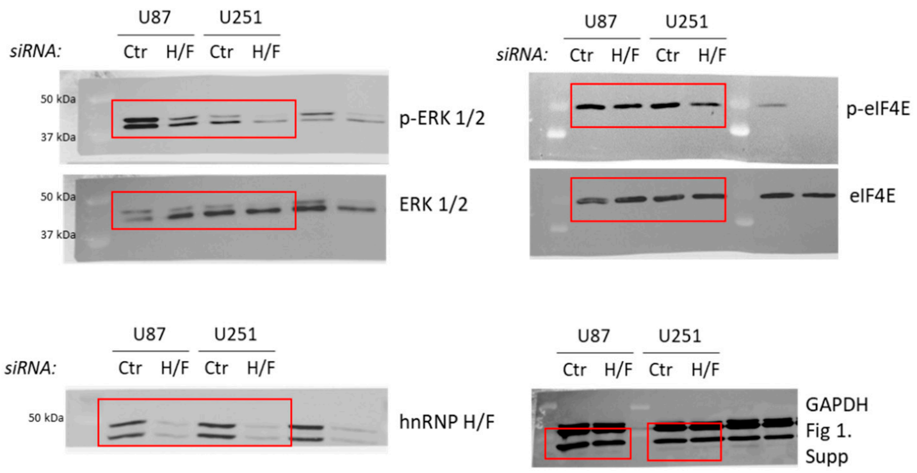

Whole Western Blot for Figure S2

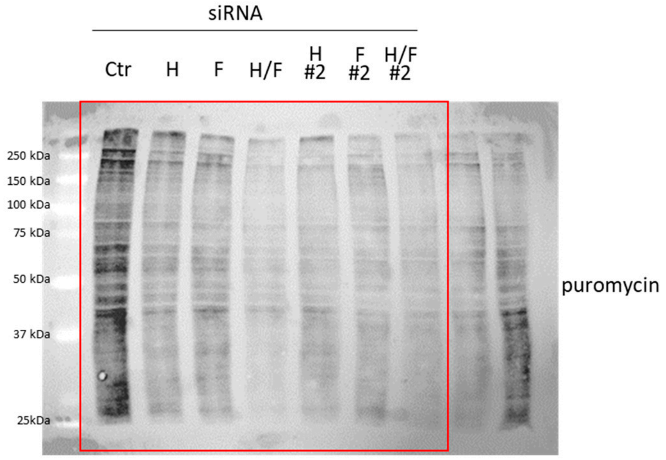

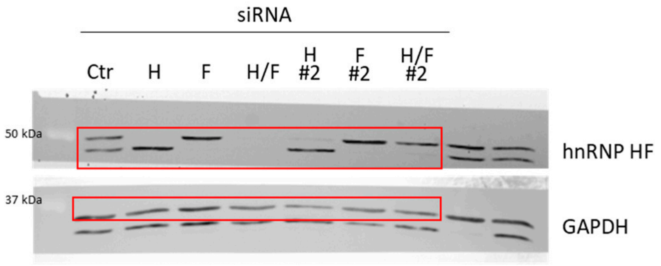

Whole Western Blot for Figure S3

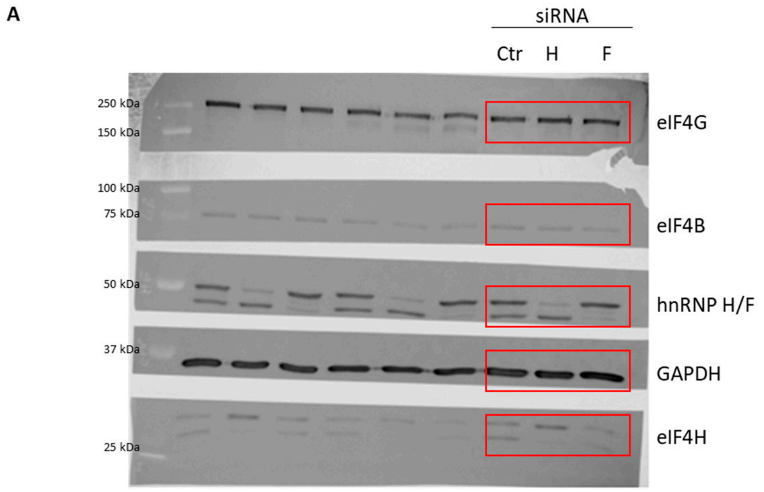

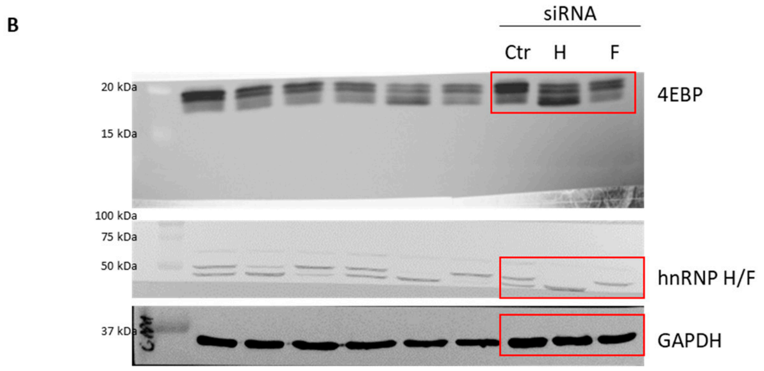

Whole Western Blot for Figure S4.

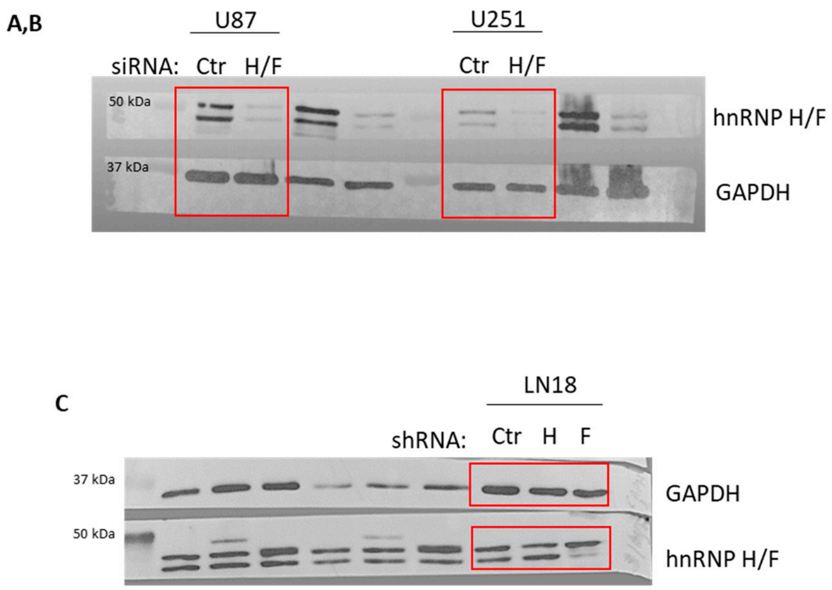

Whole Western Blot for Figure S6C

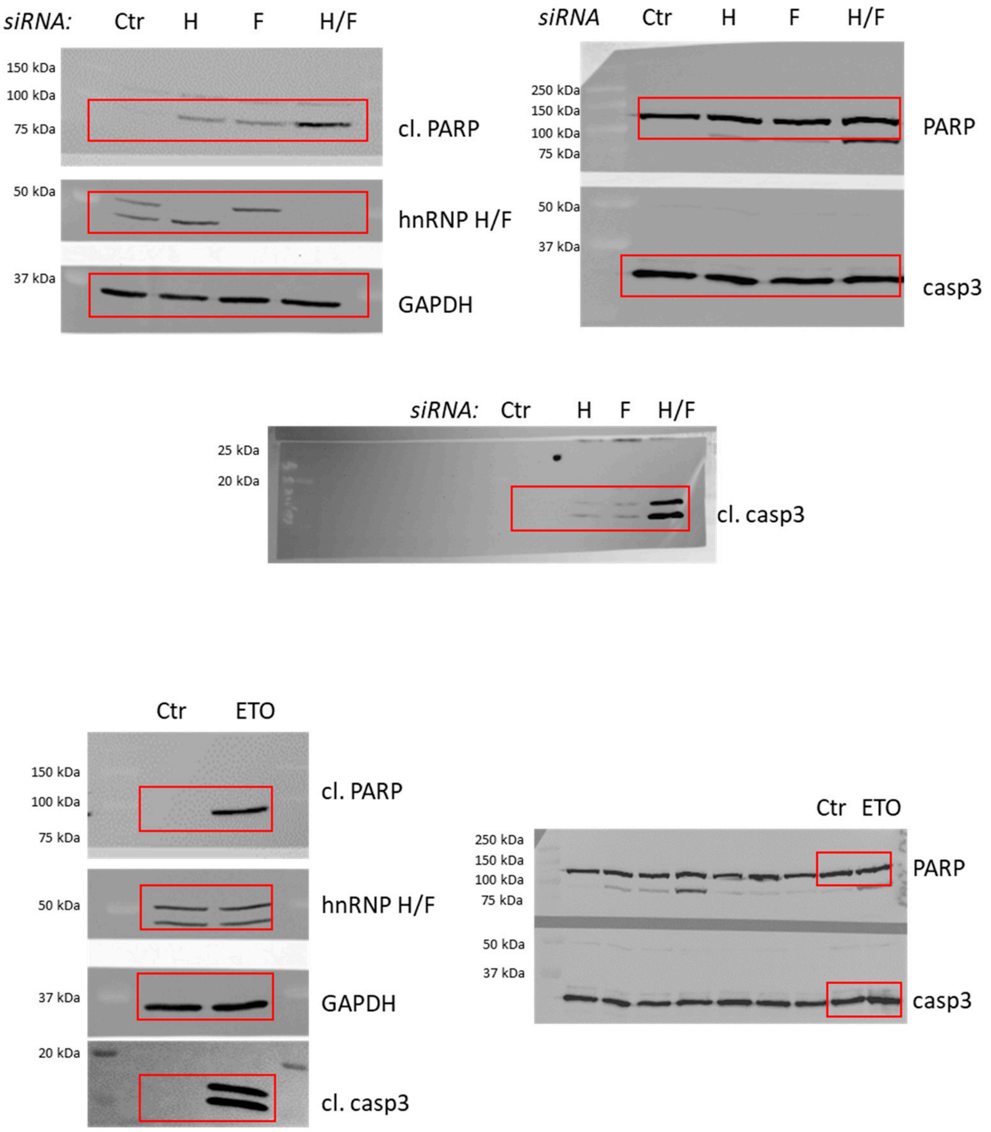

Whole Western Blot for Figure S7C

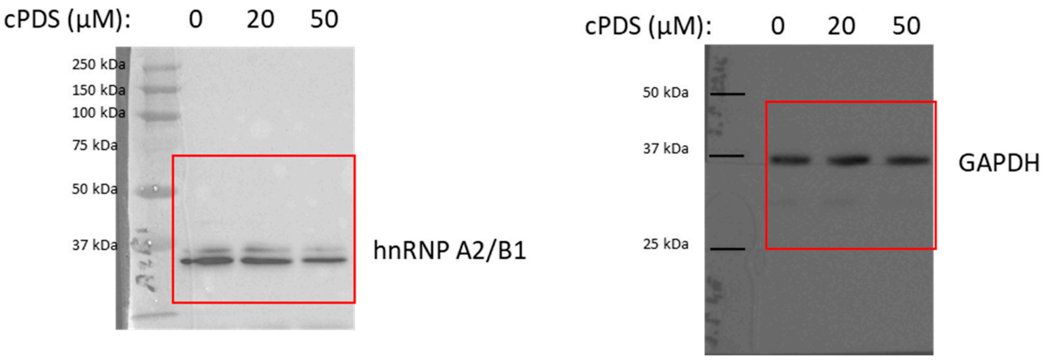

Table S1. List of siRNAs/shRNAs.

Name	siRNA/shRNA	Sequence
Control	siRNA	5′-GGUCCGGCUCCCCCAAAUG dTdT-3′
hnRNP H	siRNA	5′- GGUAUUCGUUUCAUCUACA dTdT -3′
hnRNP F	siRNA	5′- GGUGUCCAUUUCAUCUACA dTdT-3′
hnRNP H#2	siRNA	hnRNP H1: 5′- AGCGGUGGUGCUUACGAAC dTdT-3′<br>hnRNP H2: 5′- CAUGAGAGUACAUAUUGAA dTdT- 3′
hnRNP F#2	siRNA	5′- GCGACCGAGAACGACAUUU dTdT-3′
hnRNP A1	siRNA	5′- AAGGGAGGAAAUUUUGGAGGC dTdT -3′
hnRNP I	siRNA	5′- AACUUCCAUCAUUCCAGAGAA dTdT-3′
hnRNP H	shRNA	5′-AGCTGAAGTTAGAACTCATTA-3′
hnRNP F	shRNA	5′-AGCGACCGAGAACGACATTTA-3′

Table S2. List of primers used in RT-qPCR and RT-PCR.

Primer	Application	Sequence
USP1_Fw	qPCR	ACAGTCCTTAATCATTTCGGTTGA
USP1_Rv	qPCR	GGAGTTGGCATGTTTCTTGAATGT
A-Raf short_Fw	qPCR/PCR	CGGTGGTGAGTCATGGAAGC
A-Raf short_Rv	qPCR/PCR	GTATGTGCAGATGTAGGGGTCC
A-Raf full length_Fw	PCR	ATGGAGCCACCACGGGGC
A-Raf full length_Rv	PCR	CGTCTTTCGTCCCTTGATGAGTC
A-Raf_Fw	qPCR	CAAGATGGAGACGGCGGC
A-Raf_Rv	qPCR	ACCGTGCGTTGCTTGTTG
hnRNP A2/B1_Fw	qPCR	TGGAGGAGGAAGAGGAGGAT
hnRNP A2/B1_Rv	qPCR	CCATGTTCCTGCTACCACCA
HPRT_Fw	qPCR	TGCTTTCCTTGGTCAGGCAGT
HPRT_Rv	qPCR	CTTCGTGGGGTCCTTTTCACC
GAPDH_Fw	qPCR	TCACCAGGGCTGCTTTTAAC
GAPDH_Rv	qPCR	CGAGATCCCTCCAAAATCAA
hnRNP H_Fw	PCR	AAAATGGGGCTCAAGGTATTCG
hnRNP H_Rv	PCR	GCTATTTCCTGTGAAGCAAACTGC
hnRNP F_Fw	PCR	TCCAAAGACAGGGCCAATATG
hnRNP F_Rv	PCR	GCATCACCTGGCTGCTATAC
GAPDH_Fw	PCR	TGTCGCTCTTGAAGTCAGAGGAGA
GAPDH_Rv	PCR	AGAACATCATCCCTGCCTCTACTG
